# Functional Characterization of Antibodies against *Neisseria gonorrhoeae* Opacity Protein Loops

**DOI:** 10.1371/journal.pone.0008108

**Published:** 2009-12-01

**Authors:** Jessica G. Cole, Ann E. Jerse

**Affiliations:** Department of Microbiology and Immunology, F. Edward Hébert School of Medicine, Uniformed Services University of the Health Sciences, Bethesda, Maryland, United States of America; University of British Columbia, Canada

## Abstract

**Background:**

The development of a gonorrhea vaccine is challenged by the lack of correlates of protection. The antigenically variable neisserial opacity (Opa) proteins are expressed during infection and have a semivariable (SV) and highly conserved (4L) loop that could be targeted in a vaccine. Here we compared antibodies to linear (Ab_linear_) and cyclic (Ab_cyclic_) peptides that correspond to the SV and 4L loops and selected hypervariable (HV_2_) loops for surface-binding and protective activity in vitro and in vivo.

**Methods/Findings:**

Ab_SV cyclic_ bound a greater number of different Opa variants than Ab_SV linear_, including variants that differed by seven amino acids. Antibodies to the 4L peptide did not bind Opa-expressing bacteria. Ab_SV_ _cyclic_ and Ab_HV2_ _cyclic_, but not Ab_SV_ _linear_ or Ab_HV2 linear_ agglutinated homologous Opa variants, and Ab_HV2BD_ _cyclic_ but not Ab_HV2BD_ _linear_ blocked the association of OpaB variants with human endocervical cells. Only Ab_HV2BD_ _linear_ were bactericidal against the serum resistant parent strain. Consistent with host restrictions in the complement cascade, the bactericidal activity of Ab_HV2BD_ _linear_ was increased 8-fold when rabbit complement was used. None of the antibodies was protective when administered vaginally to mice. Antibody duration in the vagina was short-lived, however, with <50% of the antibodies recovered 3 hrs post-administration.

**Conclusions:**

We conclude that an SV loop-specific cyclic peptide can be used to induce antibodies that recognize a broad spectrum of antigenically distinct Opa variants and have agglutination abilities. HV_2_ loop-specific cyclic peptides elicited antibodies with agglutination and adherence blocking abilities. The use of human complement when testing the bactericidal activity of vaccine-induced antibodies against serum resistant gonococci is also important.

## Introduction

Gonorrhea is the second most commonly reported disease in the United States with over 350,000 cases reported in 2006 [1] and over 62 million estimated annual cases worldwide [2]. The gonococcus colonizes many mucosal sites, including the cervix, urethra, rectum, and pharynx. Ascended reproductive tract infections are the major source of the morbidity and mortality associated with this pathogen. Ascended infection occurs in 10–20% of cervical infections, and can lead to pelvic inflammatory disease (PID) and the associated complications of involuntary infertility, ectopic pregnancy, and chronic pelvic pain [3]. Gonorrhea is also a co-factor for transmission of the human immunodeficiency virus [4]. The public health cost of gonococcal infections is significant; over 77 million dollars were spent in the U.S. in the year 2000 on the diagnosis and treatment of acute gonorrhea and post-infection sequelae in patients 15–24 years of age [5]. The rapid emergence of antibiotic-resistant strains [6] underscores the importance of identifying new preventive measures against gonorrhea as illustrated by the recent removal of fluoroquinolones from recommended treatments [7].

The development of a gonorrhea vaccine is challenged by the lack of known correlates of protection. Repeat infections are common even with the homologous strain [8] or serotype [9], [10], although evidence of partial immunity has been reported [11], [12]. *N. gonorrhoeae* does not express a capsule, which is the target of several effective meningococcal vaccines. Therefore, research towards a gonorrhea vaccine has focused on other surface antigens such as outer membrane proteins. The neisserial opacity (Opa) proteins are a family of outer membrane proteins that mediate adherence to and invasion of tissue culture cells [13]. Gonococci express 8–11 antigenically distinct proteins that are encoded by separate *opa* genes [14], [15]. Mature Opa proteins are predicted to have four surface-exposed loops, namely, one semi-variable (SV) loop, two hypervariable (HV_1_ and HV_2_) loops, and one conserved (4L) loop [16]. Sequence differences in the HV regions are responsible for the antigenic identity of each Opa protein as well as slight differences in molecular weight. Each *opa* gene undergoes phase variation via a frame shift mechanism, and therefore, a single gonococcus can express no Opa proteins, one Opa protein, or multiple Opa proteins simultaneously [17], [18].

The expression of Opa proteins by *N. gonorrhoeae* appears to be important during urogenital tract infections. The majority of urethral isolates from naturally [19] and experimentally infected men [20], [21] expressed one or more Opa proteins, and in women, mostly Opa-positive isolates were recovered from the cervix during certain stages of the menstrual cycle [19]. Evidence for Opa protein expression during infection is also supported by the detection of Opa protein-specific antibodies in serum and genital secretions from men and women with uncomplicated urogenital tract infections, PID, or disseminated gonococcal infection [22], [23]. The presence of antibodies to multiple Opa proteins is associated with a reduced risk of PID in commercial sex workers [24], and therefore, Opa proteins may be protective vaccine antigens.

While the HV loops are highly variable among Opa proteins, the SV and 4L loops are relatively and highly conserved, respectively and could be targeted in a vaccine. Immunization with whole Opa proteins may prevent generation of high levels of antibodies against the conserved loops due to the immunodominance of the HV loops [25]. Additionally, Opa-mediated interactions with CEACAM1 on B and T lymphocytes during immunization may decrease the effector functions of these immune cells and thus prevent a robust vaccine-induced immune response [26], [27], although this hypothesis was not supported experimentally [28]. To avoid these potential pitfalls and to test whether the SV and 4L loops might carry broadly reactive, protective epitopes, here we utilize peptide-based immunization strategies to generate antibodies against the SV and 4L loops. Both linear and cyclic peptides were used to generate antibodies based on evidence that cyclic peptides induced bactericidal, conformation-dependant antibodies against meningococcal outer membrane proteins [29]. Opa loop-specific antibodies were tested for specificity, surface-binding, in vitro activity against *N. gonorrhoeae* and the capacity to protect female mice from experimental genital tract infection when delivered topically.

## Results

### Antibodies against the SV Loop and 4L Loop Are Broadly Reactive

**Figure 1:**
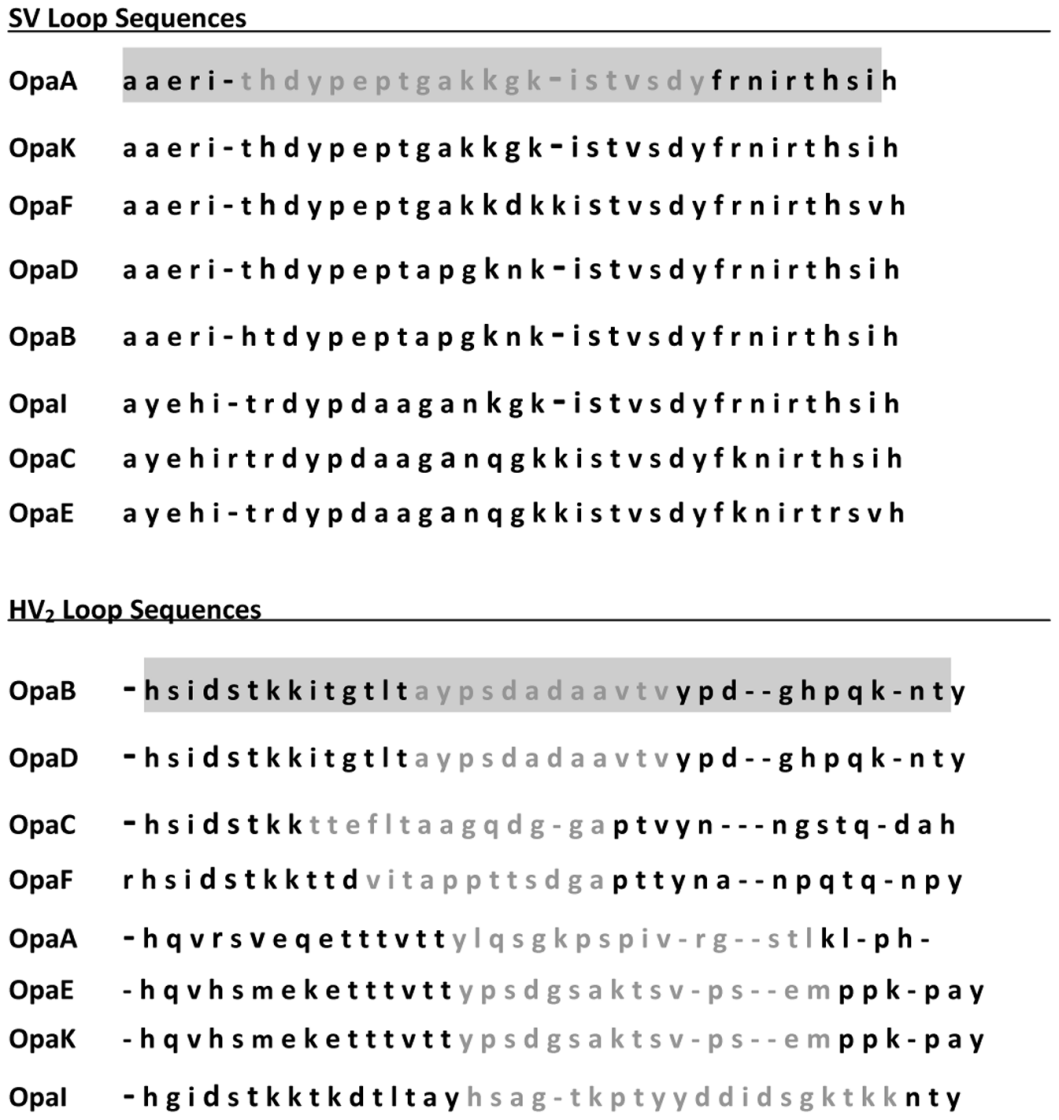
Conservation of SV and HV_2_ loop sequences. The predicted amino acid sequences of the SV and HV_2_ loops of the 8 Opa proteins of strain FA1090 are shown. The sequences of the 36-mer cyclic peptides used to generate SV- and HV_2BD_-specific antisera are highlighted in grey. The sequences of the linear peptides used to generate affinity-purified rabbit antibodies against the SV and HV_2_ loops are shown in light grey font.

**Figure 2:**
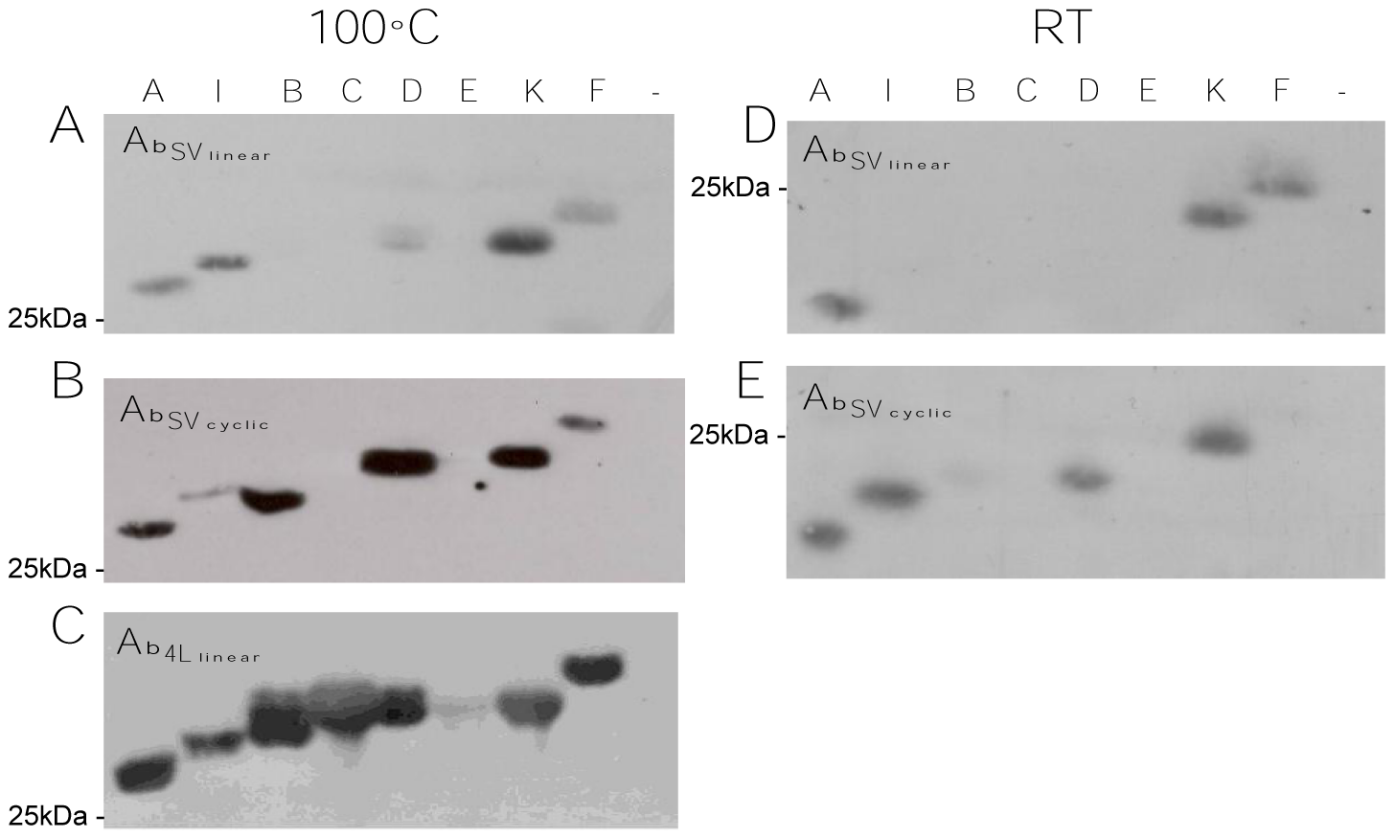
Specificity of antibodies against the conserved Opa loops. Cell lysates of an Opa-negative variant and each of the 8 Opa–positive variants of strain FA1090 were incubated at RT or 100°C prior to fractionation and incubated with (**A and D**) Ab_SV linear_ (1∶75,000), (**B and E**) Ab_SV_ _cyclic_ (1∶6,000), and (**C**) Ab_4L__linear_ (1∶6,000). The broader reactivity of Ab_SV_ _cyclic_ compared to Ab_SV linear_ is clearly shown whether native or denatured Opa proteins are analyzed. Ab_4L__linear_ is even more broadly reactive in that it recognizes all denatured Opa proteins well except OpaE. None of the antibodies recognize a protein in the Opa-negative lane (−). The location of a 25 kDa molecular weight marker is indicated, and the denatured Opa proteins migrated at a slightly higher molecular weight than those incubated at RT, which is consistent with well characterized heat modifiable nature of these proteins. The Ab_4L linear_ immunoblot was kindly provided by Dr. Amy Simms.

The 4L loop sequence of strain FA1090 is highly conserved with only a single amino acid difference among the 8 Opa proteins expressed by this strain. The SV loop is relatively well conserved with the OpaA and OpaK proteins sharing the same SV loop sequence and only 2–12 amino acid variations among the other 6 proteins (Figure 1). To test the hypothesis that antibodies against the 4L and SV loops would be broadly reactive, we examined the specificity of affinity-purified rabbit polyclonal antibodies against linear peptides that correspond to the OpaA/K SV (Ab_SV_ _linear_) and 4L (Ab_4L__linear_) loop sequences by western blot. Ab_4L__linear_ strongly recognized all of the Opa proteins of strain FA1090 except OpaE (Figure 2C) as reported previously [30]. Ab_SV_ _linear_ bound strongly to denatured OpaA and OpaK, and also to OpaF and OpaI, which have an SV loop that is predicted to differ from the OpaA/K SV sequence by 2 and 7 amino acids, respectively. Ab_SV_ _linear_ weakly recognized OpaD, which is predicted to differ from the OpaA/K SV loop by 4 amino acids. When room temperature-treated samples were analyzed, Ab_SV_ _linear_ recognized only OpaA, OpaK and OpaF. OpaD and OpaI proteins were not recognized. Ab_SV_ _linear_ did not recognize OpaB, which differs by 6 amino acids from the target peptide or OpaC or OpaE under either condition, which differ from the OpaA/K SV sequence by 11–12 amino acids (Figures 2A and D).

We also immunized mice with cyclic peptides that correspond to a longer surface-exposed region of the OpaA/K SV loop that does not include any of the predicted transmembrane regions (Figure 1) to increase the likelihood of generating conformation-dependent antibodies [29]. The resultant antiserum, Ab_SV_ _cyclic_, was more broadly reactive than Ab_SV_ _linear_ and recognized all but 2 Opa proteins of strain FA1090 when denatured samples were analyzed (Figure 2B). Ab_SV_ _cyclic_ recognized Opa proteins with up to 7 amino acid differences compared to the cyclic SV peptide, but not OpaC or OpaE, which are the most divergent from OpaA/K in the SV region (Figure 1). Ab_SV_ _cyclic_ recognized OpaA, OpaK, OpaD and OpaI proteins when RT-treated samples were used.

### SV-Specific but Not 4L-Specific Antibodies Bind the Bacterial Surface

**Figure 3:**
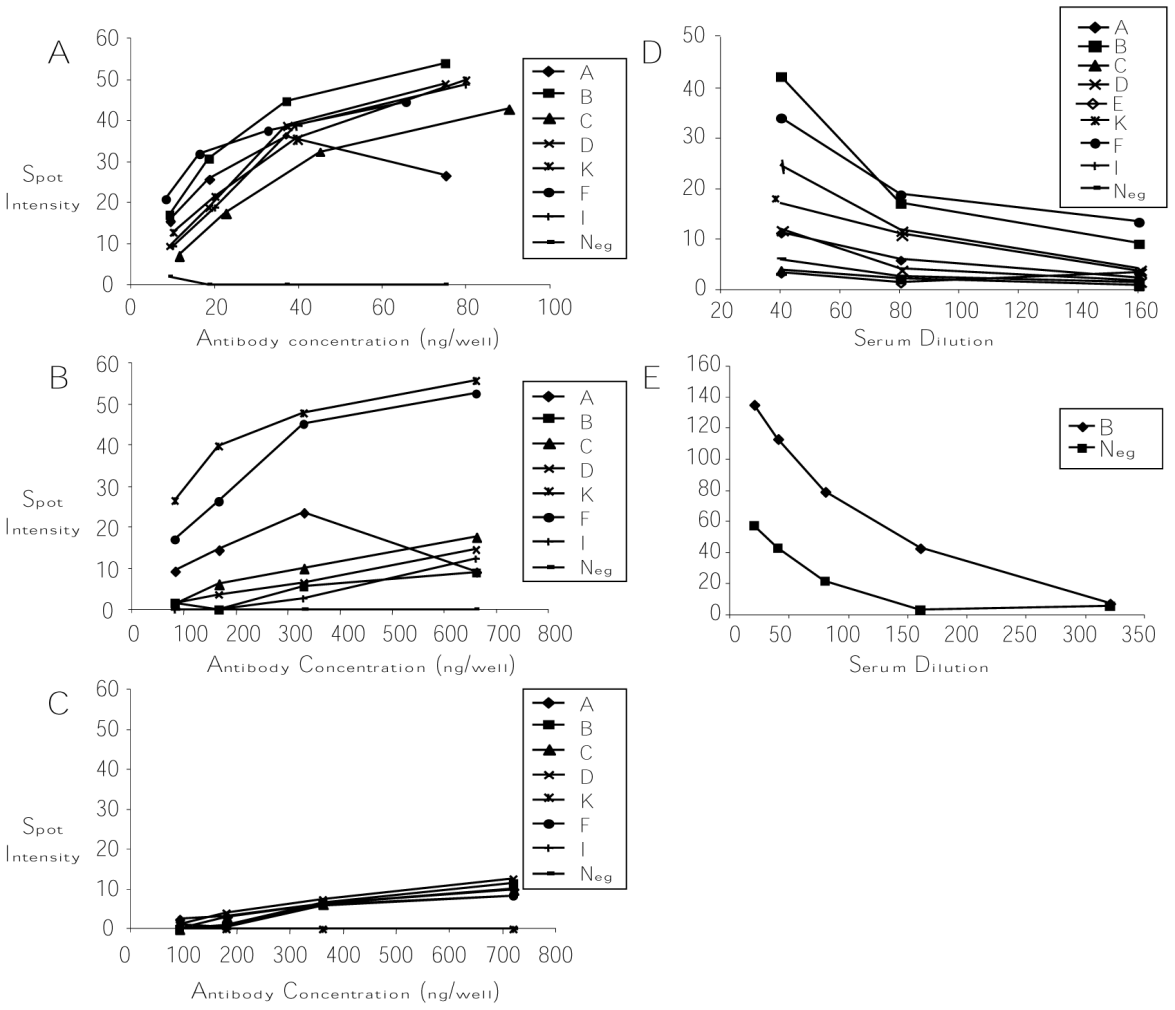
Detection of antibody surface-binding by the SBI assay. Opa-specific antibodies or antisera were serially diluted and incubated with defined Opa variants spotted onto nitrocellulose filters and binding was detected as described in the Materials and Methods. Shown are the normalized spot intensities plotted against the concentration of affinity-purified rabbit antibodies (Ab_HV2_ _linear_, Ab_SV linear_, and Ab_4L linear_) or dilution of antisera (Ab_SV cyclic_ and Ab_HV2BD cyclic_): (**A**) Ab_HV2_ _linear,_ (**B**) Ab_SV_ _linear_, (**C**) Ab_4L__linear_ (**D**) Ab_SV_ _cyclic,_ (**E**) Ab_HV2BD_ _cyclic_. All results shown are representative of at least two independent experiments. None of the antibodies bound Opa-negative gonococci. The results for Ab_HV2BD_ _linear_ when tested against Opa-negative variants are shown in panel A as an example.

The semi-quantitative surface-binding immunoblot (SBI) assay, which utilizes whole gonococci, was used to compare the concentration of antibodies needed for detectable binding to the different Opa variants of strain FA1090. Affinity-purified rabbit antibodies against linear HV_2_ loop peptides (Ab_HV2 linear_) bound homologous Opa variants in a dose-dependent manner (Figure 3A) and not heterologous or Opa-negative variants. Ab_SV_ _linear_ bound only OpaA, OpaK and OpaF variants (Figure 3B), which is consistent with western blot results against unheated lysates in which native conformations are preserved. Approximately 10-fold higher concentrations of Ab_SV linear_ were required to detect binding to whole bacteria compared to Ab_HV2_ _linear_ (compare Figures 3A and 3B). No specific binding was seen with Ab_4L linear_ at any concentration tested (Figure 3C).

The reactivity of the mouse antiserum against the cyclic SV loop peptide in the SBI assay mirrored the broader reactivity observed with this antiserum on western blots. Ab_SV_ _cyclic_ bound intact OpaA, OpaB, OpaD, OpaF, OpaK and OpaI variants above background in the SBI assay at a dilution of 1∶40, while OpaC, OpaE, and Opa-negative variants were not recognized by Ab_SV_ _cyclic_ (Figure 3D). We also tested mouse antisera against a cyclic peptide that corresponds to the HV_2_ loop of OpaB/D. As predicted, Ab_HV2BD_ _cyclic_ bound to OpaB variants better than Opa-negative variants (Figure 3E). Consistent with the greater surface exposure of the HV_2_ loop, a 5-fold higher dilution of Ab_HV2BD_ _cyclic_ produced a spot of similar intensity when tested against OpaB variants as that of the Ab_SV_ _cyclic_ antiserum (compare Figures 3D and 3E).

IFA staining was performed as a second measure of surface-binding. Consistent with the results obtained by SBI, Ab_SV_ _linear_ (2.2 µg/mL) bound OpaA variants as well as OpaK and OpaF-expressing gonococci, but none of the other Opa variants. Ab_SV_ _cyclic_ bound the same set of Opa variants as recognized in the SBI assay, but not OpaC or Opa-negative variants (Table 1). As predicted from the SBI results, mouse antiserum against the cyclic HV_2BD_ peptide bound OpaB variants at a higher dilution (1∶100) than antiserum against the cyclic SV loop peptide (1∶30). Ab_4L__linear_ did not bind any Opa-positive gonococci at concentrations of 1.2 µg/mL or 2.4 µg/mL (data not shown), a result that confirms the negative SBI results with these antibodies (Table 1).

**Table 1:** Surface-binding of SV loop-specific antibodies as assessed by SBI and IFA.

	Ab_SV_ _linear_ a		Ab_SV_ _cyclic_ b	
	IFA	SBI	IFA	SBI
**OpaA**	+	+	+	+
**OpaB**	−	−	+	+
**OpaC**	−	−	+/−	−
**OpaD**	−	−	+	+
**OpaE**	nt^c^	nt^c^	nt^c^	−
**OpaF**	+	+	+	+
**OpaK**	+	+	+	+
**OpaI**	−	−	+	+

a affinity-purified polyclonal rabbit antibodies.

b mouse antisera.

c not tested.

In summary, the IFA and SBI results were identical and confirmed the predicted surface-exposure of the SV and HV_2_ loops. The need for increased concentration of SV loop-specific antibodies to detect surface binding suggests the SV loop is less accessible than the HV_2_ loop. We also conclude that the fourth loop may not be accessible despite its predicted surface-exposure. Alternatively, the linear 4L peptide may not induce antibodies that recognize conformational epitopes present in the native protein. Finally, we also demonstrated that the cyclic SV peptide induced more broadly reactive antibodies compared to a linear peptide.

### Bactericidal and Agglutination Activities

We next investigated whether cyclic or linear peptide-derived antibodies against the SV or HV_2_-loops would be bactericidal. Disappointingly, Ab_SV_ _linear_ were not bactericidal against any of the variants tested at concentrations ranging from 4.5–120 µg/mL and the use of cyclic peptides to induce bactericidal antibodies against the SV or HV_2BD_ loops was also not successful when a dilution as low as 1∶9 was tested. Analysis of mouse antisera to cyclic loop peptides showed IgG2a>IgG1>IgG2b>IgG3. In contrast to mouse antisera against cyclic peptides, rabbit antibodies against the linear HV_2BD_ peptide, Ab_HV2BD linear_, were bactericidal against OpaB variants with a bactericidal_50_ concentration of 40.4 µg/mL. Antibodies against the HV_2_ loop of Opa I, Ab_HV2I_ _linear_, also demonstrated bactericidal activity against a homologous variant with a bactericidal_50_ concentration of 26 µg/mL. No other antibodies against HV_2_ linear peptides were bactericidal against the corresponding homologous Opa variant.

**Figure 4:**
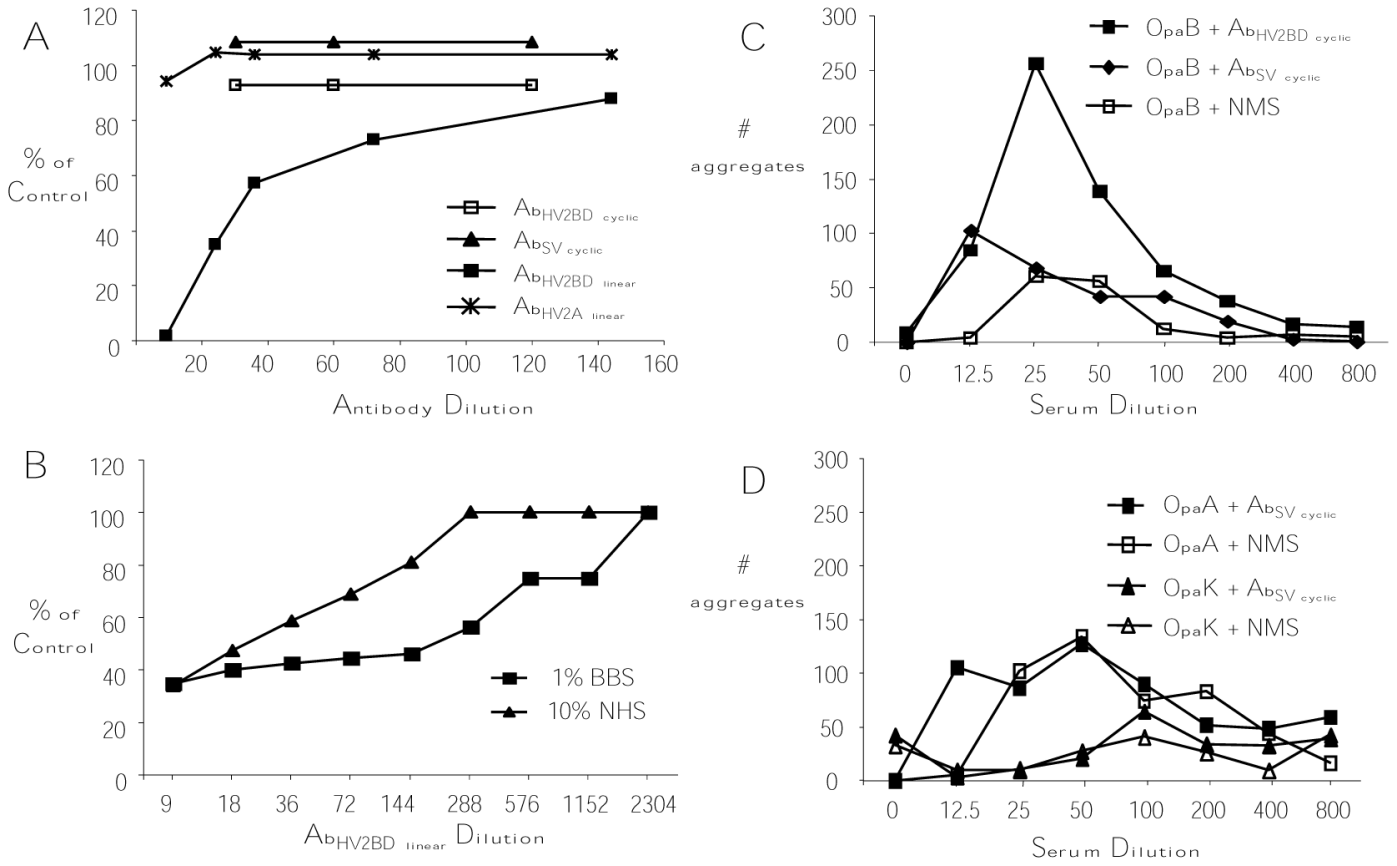
Bactericidal and agglutination activities. The bactericidal activity of SV and HV_2_ loop-specific antibodies was measured against (**A**) OpaB variants of the serum sensitive strain FA1090_F62por 5–8_ in the presence of 1% NHS and (**B**) the constitutively OpaB-expressing strain of FA1090 (SR) incubated with Ab_HV2BD linear_ in the presence of NHS or BBS. Results are expressed as the percent of bacteria recovered compared to wells with no antibody present. Only Ab_HV2BD_ _linear_ was bactericidal against the OpaB-expressing serum sensitive strain. The use of a non-human complement source (BBS) resulted in ∼8-fold greater bactericidal activity for Ab_HV2BD linear_ against serum resistant (wild type) OpaB-expressing variants. Ab_HV2BD linear_ was not bactericidal when HI-NHS and HI-BBS were used (data not shown). Agglutination activity was measured as the average number of bacterial aggregates following incubation of (**C**) OpaB variants with increasing dilutions of Ab_HVBD_ _cyclic_, Ab_SV_ _cyclic_, or NMS and (**D**) OpaA or OpaK variants with Ab_SV_ _cyclic_ or NMS. Antibodies raised to the cyclic SV peptides showed agglutinating activity except when tested against OpaK variants.

Strain FA1090 is inherently resistant to the bactericidal activity of NHS due to the binding of the complement regulatory protein human C4b-binding protein (hC4BP) to its porin [31]. This interaction is host-restricted [39]. Therefore, to better mimic events that might occur when testing the activity of these antibodies in the mouse infection model, we utilized strain FA1090_F62__por5–8,_ which produces a recombinant hybrid porin that does not bind hC4BP and is thus serum sensitive. As observed with wild type FA1090 bacteria, Ab_HV2BD_ _linear_ but not the Ab_HV2BD_ _cyclic_ or Ab_SV_ _cyclic_ mouse antisera was bactericidal against OpaB variants of strain FA1090_F62__por5–8_ in the presence of NHS. Control antibodies Ab_HV2A linear_, which like Ab_HV2BD_ _linear_ are affinity-purified rabbit polyclonal antibodies, were not bactericidal for OpaB variants of this serum sensitive strain (Figure 4A). To further investigate this host restriction, we also compared NHS with BBS as a nonhuman complement source. For these experiments we utilized a constitutive OpaB-expressing derivative of wild type strain FA1090 and a strain in which all the *opa* genes have been inactivated. Consistent with the host restriction for hC4BP, Ab_HV2BD linear_ exhibited 8-fold higher activity against the OpaB-expressing strain when BBS was used (bactericidal_50_ titers 1∶36 (28 µg/mL) with NHS versus 1∶288 (3.5 µg/mL) with BBS) (Figure 4B). Ab_HV2BD_ _linear_ showed no bactericidal activity against the Opa-deficient strain with either complement source (data not shown).

The capacity to agglutinate bacteria may facilitate shedding of bacteria in vaginal secretions and thus may be another important effector function of antibodies. Ab_HV2BD_ _cyclic_ agglutinated OpaB variants at a titer of 1∶200 with ∼40 aggregates per 40X field compared to only ∼4 aggregates per field with the same dilution of NMS. (Figure 4C). In contrast, Ab_HV2BD_ _linear_ did not agglutinate OpaB variants at dilutions as low as 1∶2 (500 µg/mL) (data not shown). Ab_SV_ _cyclic_ agglutinated OpaA and OpaB variants but not OpaK variants at a titer of 1∶12.5 compared to NMS (Figures 4C and D) although Ab_SV_ _cyclic_ bound OpaK variants. Ab_SV_ _linear_ did not agglutinate any Opa variants tested (data not shown).

### HV_2_-Specific but Not SV-Specific Antibodies Reduce Adherence to Cultured Cervical Cells

**Figure 5:**
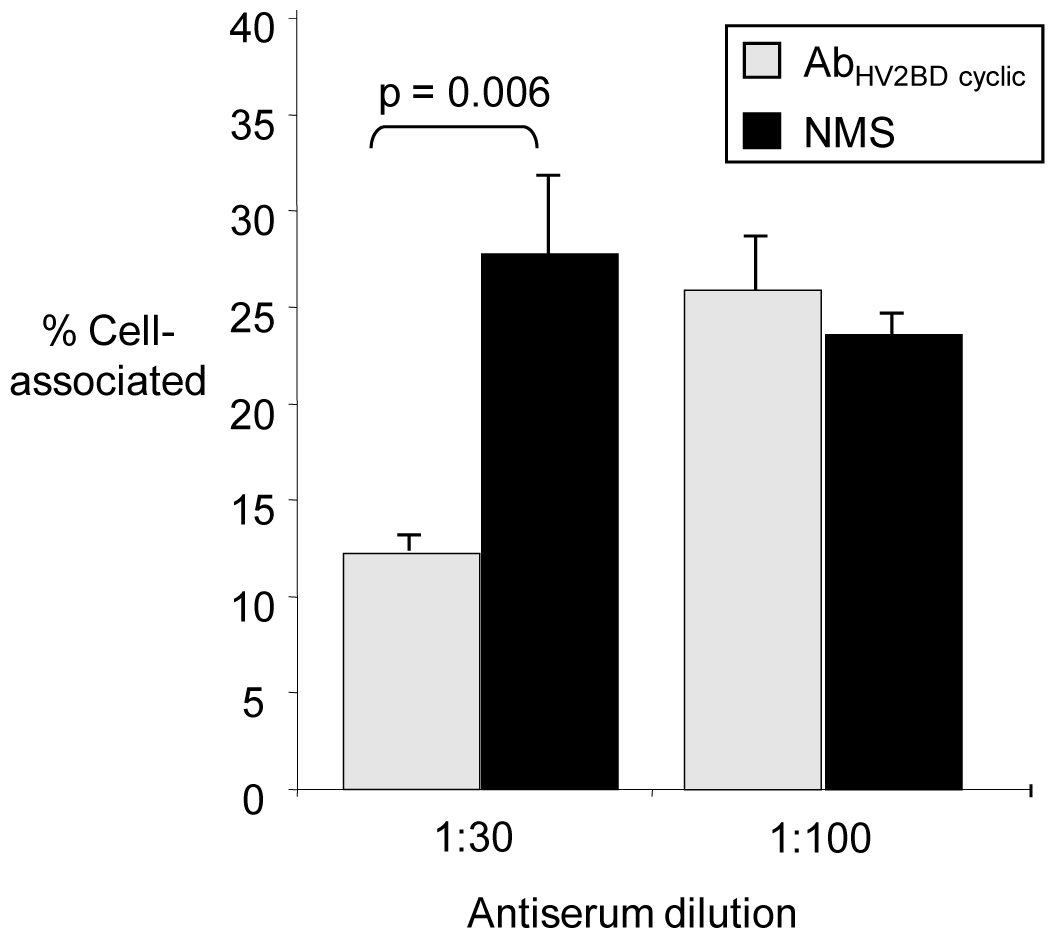
Antibody-mediated inhibition of gonococcal association with tissue culture cells. Pre-incubation with Ab_HV2BD_ _cyclic_ but not Ab_SV cyclic_ decreased the total number of ME180 cell-associated OpaB-expressing gonococci when an antiserum dilution of 1∶30 was used. Shown is the average percent of cell-associated bacteria that were preincubated with Ab_HV2BD_ _cyclic_ (test) or NMS (control) based on combined data from three independent assays. A two-tailed, unpaired Student's t-test was used to assess statistical differences between test and control wells.

Antibody-mediated inhibition of Opa-mediated adherence and invasion may also be protective. Most Opa proteins mediate invasion of human cells via binding to human carcinoemybryonic cellular adhesion molecules (CEACAMs) [13]. To test whether Opa loop-specific antibodies can block gonococcal interactions with human CEACAM-expressing endocervical cells, ME180 cells were inoculated with the constitutive OpaB-expressing strain following incubation with Ab_HV2BD linear_, Ab_HV2BD_ _cyclic_, or Ab_SV_ _cyclic_. Treatment with Ab_HV2BD_ _cyclic_ (Figure 5) but not Ab_SV_ _cyclic_ (data not shown) resulted in a dose-dependent decrease in the number of cell-associated bacteria as compared to NMS. In contrast there was no decrease in the number of cell-associated bacteria when bacteria were treated with 0.25 µg/mL or 2.5 µg/mL of Ab_HV2BD_ _linear_ versus Ab_HV2C_ _linear_, which does not bind OpaB variants (data not shown). We conclude Ab_HV2BD cyclic_, but not Ab_HV2BD linear_ block OpaB-mediated interactions with human endocervical cells.

### Passive Protection Studies

The CEACAM residues that are important for Opa-mediated adherence are not conserved in the murine CEACAM1 [30], [40], and consistent with this host restriction, we have not observed Opa-mediated adherence to two different murine epithelial cell lines (J. G. Cole et al, manuscript submitted). However, female mice can be experimentally infected with *N. gonorrhoeae* despite the absence of human CEACAMs [30], [32], and Opa-specific antibodies could prevent or reduce colonization of mice through bactericidal activity, opsonophagocytic uptake, or agglutination with subsequent shedding. Additionally, because selection for Opa expression occurs during experimental infection of BALB/c mice for reasons that are not known [30], Opa-specific antibodies may block other Opa-mediated functions [38]. We therefore tested the capacity of Opa loop-specific antibodies to reduce gonococcal colonization of BALB/c mice when administered vaginally as done in protection studies for other sexually transmitted pathogens [37], [41]–[43].

**Figure 6:**
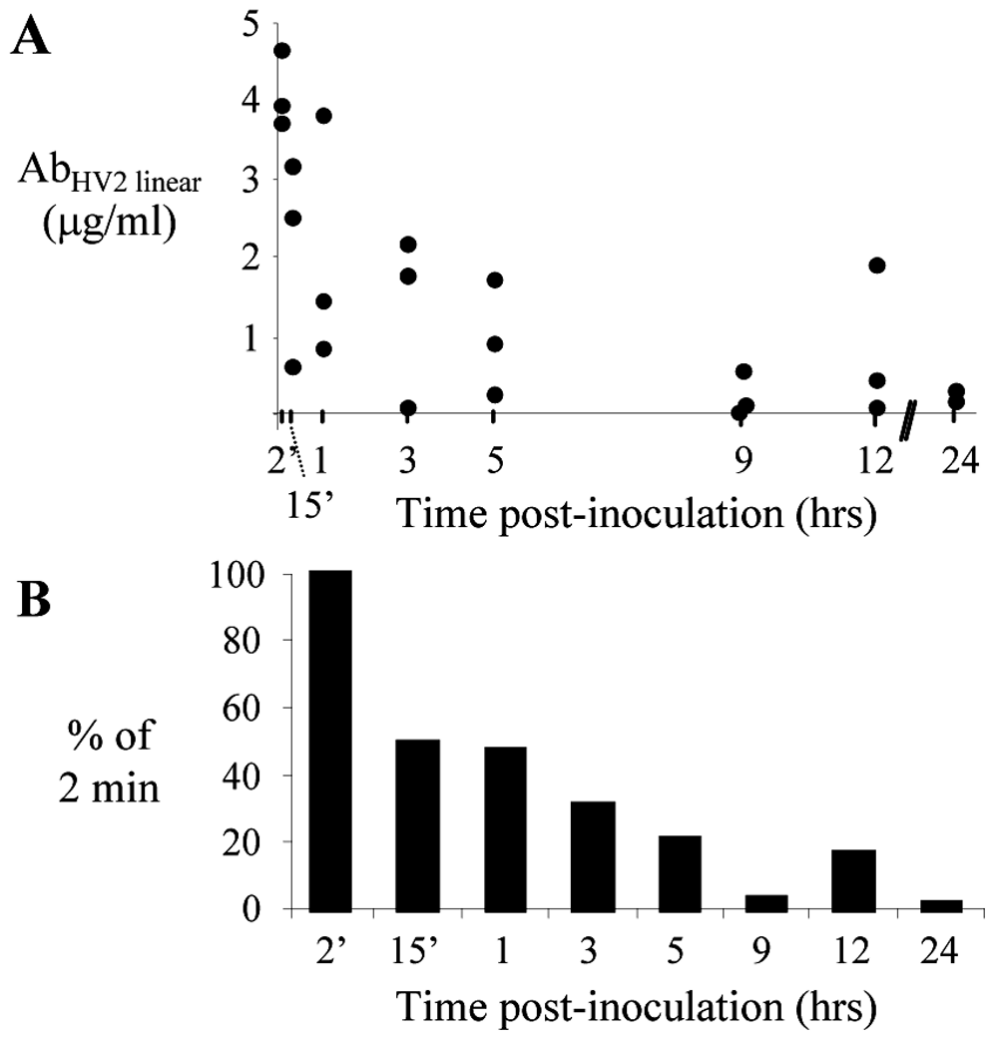
Duration of topically applied antibodies in the vagina. A fifty percent reduction in the concentration of topically applied rabbit antibodies was detected in vaginal washes collected 1 hr post-inoculation compared to a 2 min time point. Antibody levels decreased further over time. The limit of detection was 7.8 ng/ml. (**A**) Concentration of rabbit IgG (µg/ml) in mouse vaginal washes was determined in duplicate by capture ELISA. (**B**) The percent of antibody remaining relative to the average 2 minute value is shown for each time point. Results are combined from two experiments and each animal was used for a single time point (n = 2–3 mice per time point).

First, to determine how long antibodies remain in the vagina after topical administration, groups of mice were inoculated vaginally with 20 µl of PBS containing 10 µg of Ab_HV2_ _linear_, and the amount of rabbit IgG in vaginal washes was measured over time. A 50% decrease in the amount of rabbit IgG recovered was observed between 2 and 15 min post-inoculation. Antibody levels were maintained for at least another 45 min, after which a gradual decline was observed over the next 4 hours. Low levels of antibody were detected in all but one mouse at 9, 12, and 24 hrs post-inoculation (Figure 6). No rabbit IgG was detected in vaginal washes from untreated control mice and there was no correlation between persistence of antibody and the stage of estrous at the time of inoculation. Based on these data we concluded that antibody must function within the first 24 hrs to be effective, and thus chose to analyze colonization loads only at early time points (days 1, 2, and 3 post-inoculation). We also chose a challenge dose of 10^3^ CFU for subsequent protection experiments based on pilot experiments that showed this dose resulted in colonization by strain FA1090 for at least three days in 85% percent of mice.

**Figure 7:**
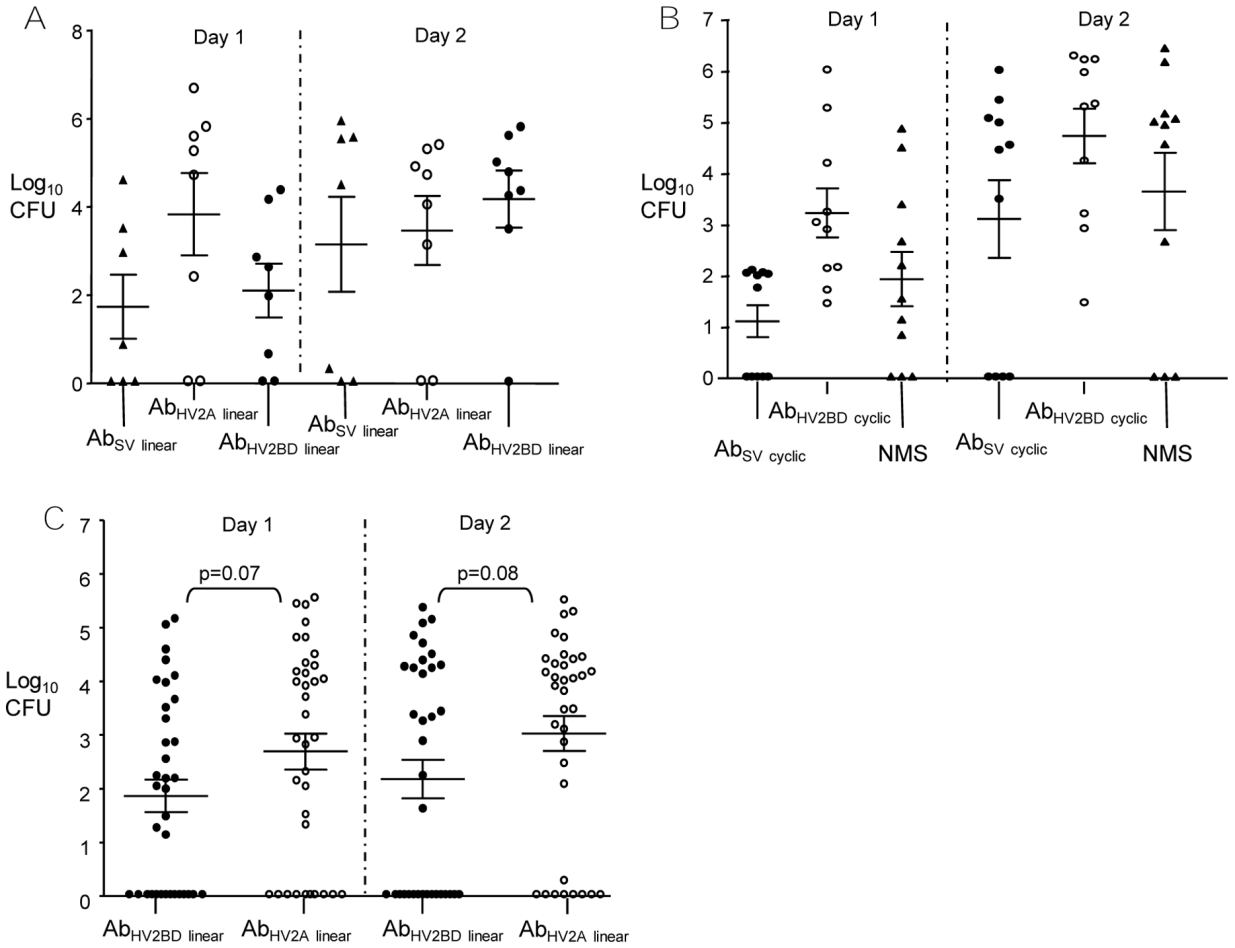
Passive protection experiments. Mice were inoculated with OpaA or OpaB variants that were preincubated with antibodies against linear or cyclic Opa SV or HV_2_-loop sequences. (**A**) OpaA variants with Ab_SV_ _linear_, Ab_HV2A linear_, or Ab_HV2BD linear_. There was no difference in the number of gonococci recovered from mice in the Ab_SV_ _linear_ and Ab_HV2A linear_ test groups compared to the Ab_HV2B linear_ control group on days 1 or 2 post-inoculation (p≤0.14). (**B**) OpaB variants with Ab_HV2BD_ _cyclic_, Ab_SV_ _cyclic_ or NMS. There was no difference in the number of gonococci recovered from mice treated with Ab_SV_ _cyclic_ compared to the control (NMS) group (p≤0.20) on days 1 or 2 post-inoculation. (**C**) OpaB variants with Ab_HV2BD linear_ and Ab_HV2A linear_. Shown are combined data from three independent experiments that showed no difference in the number of bacteria recovered from each group on days 1 and 2 post-inoculation at the level of p = 0.07 and p = 0.08, respectively (n = 7–15 mice/group in each experiment; total number = 35 mice/group). Symbols indicate a single animal and horizontal bars indicate the group mean with the SEM shown. Statistical differences were analyzed by a two-tailed Student's *t* test.

We first tested Ab_SV_ _linear_ to address our main objective of developing broadly-reactive protective antibodies against *N. gonorrhoeae*. Mice were inoculated with 10^3^ OpaA variants that were preincubated with Ab_SV_ _linear_, Ab_HV2A linear_, or Ab_HV2BD linear_. OpaA variants were used since Ab_SV_ _linear_ bound OpaA variants (Figure 3B and Table 1). There was no significant difference in the number of gonococci recovered on days 1 and 2 post-inoculation when results from the Ab_SV_ _linear_ and Ab_HV2A linear_ groups were compared to the Ab_HV2BD linear_ control group (Figure 7A). We also tested Ab_SV_ _cyclic_, which may recognize conformational epitopes among Opa proteins compared to Ab_SV_ _linear_. Ab_SV_ _cyclic_ bound OpaB variants well in the SBI and IFA assay, and we therefore assessed the capacity of Ab_SV_ _cyclic_ and Ab_HV2BD_ _cyclic_ to passively protect mice from OpaB variants. Incubation of OpaB variants with Ab_HV2BD_ _cyclic_ or Ab_SV_ _cyclic_ did not result in a significant difference in colonization load (Figure 7B) or percent of test mice colonized on day 3 compared to the NMS control group (data not shown). In summary, despite binding to the bacterial surface, antibodies against the SV loop do not reduce *N. gonorrhoeae* colonization, even when a cyclic peptide is used to better mimic the loop conformation.

None of the HV2-specific antibodies that we tested as potential positive controls in studies with SV loop-specific antibodies showed protection. Results from initial pilot studies suggested inoculation of mice with 10^3^ CFU of OpaB variants incubated with 5 µg Ab_HV2BD_ _linear_ would be protective in passive protection studies. Unlike Ab_HV2BD cyclic_, Ab_HV2BD linear_ were bactericidal, and therefore, we performed a larger scale experiment to test the capacity of Ab_HV2BD linear_ to protect mice challenged with OpaB variants. Significantly fewer bacteria were recovered from mice in the Ab_HV2BD_ _linear_ test group compared to mice for which Ab_HV2A_ _linear_ was used as a negative control (p≤0.03) on days 1 and 2 post-inoculation. However, this difference was not observed in two subsequent experiments and statistical analysis of combined data from all three experiments showed no significant decrease in recovery of gonococci from mice inoculated with Ab_HV2BD linear_-treated OpaB variants on days 1 and 2 post-inoculation compared to Ab_HV2A_ _linear_-treated bacteria (p = 0.07 and p = 0.08, respectively) (Figure 7C). There was also no difference in the number of mice colonized in each group with 60% of the Ab_HV2BD_ _linear_ and 71% of the Ab_HV2A linear_-treated mice infected on day 1 (p = 0.45). We considered the possibility that subpopulations of gonococci that express a different Opa protein than that of target variant were responsible for the colonization of test groups. However, the distribution of Opa phenotypes of vaginal isolates on day 1 did not differ significantly from that of the inoculum in any experiment. We conclude that none of the HV_2_-specific antibodies tested are protective against *N. gonorrhoeae* colonization, including antibodies with bactericidal activity, and the lack of effectiveness of these antibodies in vivo is not due to escape variants establishing infection.

## Discussion

*N. gonorrhoeae* is a highly successful Gram-negative bacterium that is noted for the antigenic variability of its surface and the frequency by which it causes repeat infections. Opa proteins are expressed during infection and have two conserved loops, the SV and 4L loops, which could be targeted in a vaccine. Here we analyzed antibodies against peptides that correspond to the SV and 4L loops for the capacity to bind to gonococci that express different Opa proteins and for correlates of antibody-mediated protection. Antibodies against linear or cyclic peptides that correspond to the SV loop recognized intact gonococci as assessed by two different methods, and promisingly, antibodies generated against cyclic SV peptides bound the surface of 6 of the 8 Opa variants of strain FA1090, including Opa variants with 7 amino acid differences from the target peptide. In contrast, 4L-specific antibodies did not bind the bacterial surface. The cyclic SV loop peptide also induced antibodies with agglutination ability, while the SV linear peptide did not. The broader reactivity and agglutinating ability of Ab_SV cyclic_ may be explained by the fact that the cyclic SV peptide was longer (36 amino acids) than the linear peptides (20 amino acids) and included more conserved regions of the loop. Cyclic peptides may therefore carry more conserved epitopes, T-cell epitopes, and possibly conformational epitopes that are shared by Opa proteins with a less related primary sequence. Interestingly, bactericidal activity was only exhibited by antibodies against linear peptides. This result was in contrast to the demonstration that cyclic peptides were more successful for inducing bactericidal antibodies against the class 1 protein of *N. meningitidis* [29].

With regard to the difference in bactericidal activity between Ab_HV2BD linear_ and Ab_HV2BD cyclic_, one should consider the fact that the antibodies were produced in different animal species and that Ab_HV2BD linear_ were high titer affinity-purified antibodies. Antibodies generated by the linear or cyclic SV loop peptides were not bactericidal, however, even when rabbit serum was used as a complement source to by-pass the host-restricted serum resistance of this strain. It is possible that a different adjuvant might improve the potential of the SV loop as a vaccine target by promoting the induction of bactericidal antibodies.

We also showed that the species used as the complement source in bactericidal assays is important when examining the bactericidal activity of vaccine-induced antibodies against SR strains of *N. gonorrhoeae*. Some P1B strains, like strain FA1090 are resistant to NHS due to the binding of human C4BP to the P1B molecule [31], which reduces activation of the classical pathway and subsequent bacteriolysis [44]. Here we demonstrated that Ab_HV2BD_ _linear_ killed OpaB variants of a SS derivative of strain FA1090 better than SR wild type OpaB variants when NHS was used as the complement source. Ab_HV2BD_ _linear_ was also more bactericidal against OpaB variants of strain FA1090 when rabbit serum was used. These results illustrate the importance of considering the complement source in bactericidal assays designed to predict vaccine efficacy in humans versus laboratory animals. Ideally, antibodies that are strongly bactericidal against SR strains in the presence of NHS are desired.

A vaccine-induced immune response against Opa proteins could also block Opa-mediated adherence and invasion, which has been the focus of vaccine studies on meningococcal Opa proteins. de Jonge et al. [25], [28] reported that antibodies raised against purified recombinant Opa proteins, outer membrane protein vesicles containing Opa proteins, or liposomes containing recombinant Opa proteins from *N. meningitidis* elicited an antibody response, which while not always bactericidal, blocked Opa-CEACAM interactions on tissue culture cells [25]. Here we showed Ab_HV2BD_ _cyclic_ but not SV-specific antibodies decreased the total number of bacteria associated with CEACAM-expressing endocervical cells. The inability to block adherence with SV-specific antibodies is in accordance with studies on *N. meningitidis* Opa proteins that show the SV loop is not involved in CEACAM-binding [46]. Other Opa protein functions are likely to exist, however, that could perhaps be blocked by an SV loop-specific immune response. For example, Opa-expressing gonococci are preferentially recovered from experimentally infected male volunteers [20], [21] and during lower genital tract infection of female mice [30]. The basis for this selection is not known, and the inability to detect CEACAMs on primary male urethral cells [47] and the dissimilarities between human and murine CEACAM1 [40], [48] suggest Opa proteins may play other important roles during infection [49].

Finally, SV loop-specific and selected HV_2_ loop-specific antibodies showed no protection in mice when mixed with the homologous variant prior to vaginal inoculation. We conclude the SV or HV_2_ Opa protein loops may not be effective targets for antibody-mediated protection. It is possible, however, that technical limitations may have prevented us from detecting a protective effect. The concentration of antibodies may not have been high enough and similar to that reported by Sherwood et al. [50], antibodies were gradually lost within 24 hrs post-inoculation. Clearance via opsonophagocytosis might not occur during this time frame since phagocytes are not detected in infected murine tissue until 2 to 5 days post-inoculation [38]. Systemic delivery of antibodies to the vagina has been successful for others. For example, monoclonal IgA against *Chlamydia trachomatis* was detected in mouse vaginal secretions for up to 48 hrs when delivered intraperitoneally [37], and Parr et al. [41] reported intraperitoneal administration of IgG resulted in the detection of specific IgG 48 hours later in vaginal secretions at levels equal to 3% of that found in the vaginal secretions of immunized mice. We have detected rabbit IgG in vaginal washes for as long as 60 hrs after intraperitoneal or intravenous injection of high titer rabbit polyclonal antiserum against whole gonococci (B.T. Mocca and A.E. Jerse, unpublished data). However, we did not detect gonococcal-specific antibodies in vaginal washes following intravenous injection of the affinity-purified HV_2_-specific rabbit antibodies used in this study, and we therefore chose to deliver the antibodies topically.

In summary, we have demonstrated that broadly-reactive antibodies can be generated against a relatively conserved Opa protein loop that bind to the bacterial surface and have agglutination ability. These antibodies could potentially recognize many Opa variants produced by different gonococcal strains and therefore, the use of different adjuvants or other strategies to induce high titered SV loop-specific antibodies with bactericidal activity that can be delivered systemically is warranted. The in vitro and in vivo experiments described here should be useful in the development of other vaccine antigens against gonorrhea.

## Materials and Methods

### Strains and Culture Conditions

*Neisseria gonorrhoeae* strain FA1090 [*porB1b*, streptomycin resistant, serum resistant (SR)] was originally isolated from a female patient with disseminated gonococcal infection. Strain FA1090 expresses 8 antigenically distinct Opa proteins: OpaA, OpaB, OpaC, OpaD, OpaE, OpaF, OpaI and OpaK. Frozen stocks of each Opa variant were prepared as described [30]. Stock Opa variants expressed LOS species with the same banding pattern on silver stained tricine gels (data not shown) and stocks composed of either mostly piliated or nonpiliated variants were maintained. Strain FA1090_F62por5–8_ is a serum sensitive (SS) derivative of strain FA1090 in which porin loops 5–8 were replaced with loops 5–8 of the SS strain F62 as described by Ram et al. (kindly provided by Sanjay Ram, University of Massachusetts) [31]. Strain FA1090_F62__por5–8_ is sensitive to NHS, and does not bind human C4b-binding protein (hC4BP). OpaA, OpaF, and Opa-negative variants were isolated from OpaB-expressing FA1090_F62por5–8_ bacteria after 2–3 serial passages of individual colonies that were screened by colony suspension immunoblots with HV_2_-specific antibodies as described [30], [32]. Where indicated, recombinant strains of FA1090 that express no Opa proteins or that constitutively express only OpaB were used (kindly provided by Janne Cannon, University of North Carolina). *N. gonorrhoeae* was cultured on GC agar (Difco) with Kellogg's supplement [33] and 0.2 µM Fe(NO_3_)_3_ at 37°C under 7% CO_2_. GC-VCNTS agar (GC agar with vancomycin, colistin, nystatin, trimethoprim, and streptomycin sulfate) was as described [32].

### Generation of Antibodies

Two general types of antibodies were evaluated in this study, specifically affinity-purified rabbit polyclonal antibodies against linear peptides (Ab_linear_) and mouse antisera raised against cyclic peptides (Ab_cyclic_). Ab_4L__linear_, Ab_HV2A linear_, Ab_HV2BD linear_, Ab_HV2C linear_, Ab_HV2F linear_, Ab_HV2I linear_, and Ab_HV2K linear_ antibodies were previously described [30]; here we obtained SV-loop specific rabbit antibodies (Ab_SV_ _linear_), which were generated against a linear 20 amino acid peptide (DYPEPTGAKKGKISTVSDYF) that corresponds to the SV loop of OpaA and OpaK (OpaA/K) of strain FA1090 (Figure 1). Peptide synthesis, rabbit immunizations, and affinity purification were performed by Bethyl Laboratories (Montgomery, Texas). Rabbit antibodies were dialyzed (50 kDa exclusions pore size) (Spectrum Laboratories Inc, Racho Dominguez, CA) to remove the 0.1% sodium azide that was added during preparation. We also generated mouse antisera (Ab_SV cyclic_ and Ab_BD cyclic_) to two cyclic peptides that correspond to the SV loop sequence of OpaA/K (AAERITHDYPEPTGAKKGKISTVSDYFRNIRTHSIH; 36-mer) or the HV_2_ loop sequence that is common to OpaB and OpaD (OpaB/D) (IDSTKKITGTLTAYPSDADAAVTVYPDGHPQKNTYQ; 36-mer). Cyclic peptides were synthesized by Celtek Peptides (Nashville, TN) through the addition of a disulfide bond between added terminal cysteine residues, and six week-old female BALB/c mice were immunized subcutaneously three times at three week intervals with 50 µg of peptide suspended in TiterMax Gold (Sigma Chemical Co, St. Louis, MO). Blood was collected two weeks after the final boost, and individual samples were analyzed by enzyme linked immunosorbent assay (ELISA) for peptide-specific antibody titers essentially as described [34]. Briefly, 96-well plates were coated with 5 µg of the cyclic peptide in 50 mM NaHCO_3_ (pH 9.6), and incubated with three-fold dilutions of mouse sera followed by goat anti-mouse IgG (γ chain-specific) conjugated to horseradish peroxidase (HRP) and HRP substrate (Sigma). Absorbance was read at 405 nm on an EL800 Universal Microplate Reader (Bio-Tek Instruments, Winooski, VT) and analyzed with KC Junior software (Bio-Tek Instruments). Background was set at 3 standard deviations above the average A_405_ readings of 3 wells to which no primary antibody was added. Sera with titers >1∶7,290 were pooled and frozen at −20°C. The relative levels of IgG isotypes within Opa loop-specific mouse antisera were measured with a mouse antibody isotyping kit (Bio-Rad Laboratories, Hercules, CA) as per the manufacturer's instructions.

### Immunoblots

For western blots, bacteria were suspended in lithium acetate buffer and incubated in Laemmli buffer (Sigma) containing sodium dodecyl sulfate (SDS) and β-mercaptoethanol for 10 min at 100°C to denature the samples or at room temperature (RT) to preserved native conformations. Samples were fractionated on 11.5% SDS polyacrylamide gels and transfered to polyvinylidene fluoride (PVDF) membranes. After blocking in 0.5% Tween 20, membranes were incubated with Ab_SV linear_ (1∶75,000), Ab_4L linear_ (1∶6,000), or Ab_SV_ _cyclic_ (1∶6,000), followed by goat anti-rabbit IgG HRP (1∶50,000) (Bethyl Laboratories) or anti-mouse IgG HRP (1∶50,000) (Sigma). Primary and secondary antibodies were diluted in block, and blots were washed three times in PBS with 0.05% Tween 20 after each incubation. Detection was with ECL detection reagent (Amersham Biosciences) as per the manufacturer's instructions. Attempts to utilize an ELISA with whole bacteria as the antigen to measure surface-binding were not successful due to the bacteria not adhering well enough to the wells. Therefore, a semi-quantative surface-binding immunoblot (SBI) similar to that described by Afonina et al. [35] was used to measure the binding of antibodies to intact gonococci. Bacteria of the Opa phenotype to be tested were suspended in PBS to an A_600_ of 0.20 and diluted 1∶20 in PBS. One hundred microliters (∼5×10^5^ CFU) of the final suspensions were applied to a nitrocellulose membrane via a 96-well vacuum manifold apparatus (Schleicher & Schuell, Keene, NH). The membrane was dried at RT, incubated for 30 min at 37°C, and then blocked for 1 hr in PBS with 3% BSA (Sigma). The filter was returned to the manifold and individual wells were incubated for 1 hr with 100 µL of two-fold serial dilutions of affinity-purified rabbit antibodies (Ab_SV linear_, Ab_4L_, Ab_HV2_ _linear_; range 8.2–720 ng) or mouse antisera (Ab_sv cyclic_, Ab_HV2BD cyclic_; range 1∶20–1∶320). Positive control wells were incubated with serial dilutions of rabbit polyclonal antiserum against heat-killed FA1090 bacteria (1∶4,000–1∶64,000) or the porin-specific monoclonal antibody B2E8 (1∶250–1∶4,000) (A.E. Jerse and Mary Petzke, unpublished data). All antibodies were diluted in PBS with 3% BSA, and secondary detection, washes, and exposure of the membranes to substrate were as for western blots. Spot intensities were quantified by densitometry (Image J Version 1.37v) and the mean intensity of three wells incubated without primary antibody was subtracted from that of wells with Opa-specific antibodies (test wells) or anti-whole bacteria or B2E8 antibodies (control wells). The spot intensities of the control wells were plotted against the antibody concentration (Ab_linear_, rabbit) or antiserum dilution (Ab_cyclic_, mouse), and values that fell within the linear regions of the curves were used to normalize for slight differences in the number of bacteria in each spot. Normalized data were obtained by dividing the mean intensity of the test wells by that of the appropriate control well (mouse or rabbit antibody).

### Indirect Fluorescent Antibody (IFA) Staining

Single colonies of FA1090 Opa variants were suspended in water, applied to IFA slides (Electron Microscopy Sciences, Hatfield, PA), and fixed in 100% methanol at −20°C after drying at RT. Slides were blocked for 1 hr in PBS with 0.1% immunoglobulin-free BSA (Sigma) (blocking buffer). Slides were incubated with primary antibodies for 1 hr at the following final concentrations or dilutions, which were determined empirically: Ab_HV2 linear_ (0.87–1.2 µg/mL), Ab_SV_ _linear_ (2.2 µg/mL), Ab_4L linear_ (2.4 µg/ml), and Ab_HV2 cyclic_ (1∶100) and Ab_SV cyclic_ (1∶30). Secondary antibodies were goat anti-rabbit or goat anti-mouse IgG conjugated to AlexaFluor 488 (Invitrogen, Carlsbad, CA) (1∶500) and incubations were for 30 min. All antibodies were diluted in blocking buffer and wells were washed five times with PBS after each incubation. Antibodies specific for the HV_2_ loop of each Opa variant were used as positive controls (0.87–1.2 µg/mL) in all IFA assays; polyclonal rabbit antisera against heat-killed FA1090 (1∶1,000) was used as a positive control for Opa-negative variants; negative controls were antibodies against heterologous HV_2_ loops and wells that were not incubated with primary antibodies. Slides were examined with an Olympus BX60 system microscope with a BX-FLA reflected light fluorescence attachment and Olympus U-M41001 filter. All images were obtained with a SPOT charge-coupled-device digital camera (Diagnostic Instruments Inc., Sterling Heights, MI).

### Bactericidal Assay

Bactericidal assays against wild type FA1090 Opa variants were performed in microtiter plates using 10% normal human serum (NHS) (Quidel Corporation, San Diego, CA) or 1% baby bunny serum (BBS) (AbD Serotec, Raleigh, NC) as the complement source. For assays that used strain FA1090_F62por5–8_, 1% NHS was used. These serum concentrations are >2-fold lower than that concentration that showed no killing of the target strains in the absence of added antibody as determined by Garvin et al [36]. For testing Opa loop-specific antibodies, Ab_linear_ or Ab_cyclic_ were serially diluted in minimal essential medium (MEM) (final volume 150 µl), and 50 µl of diluted NHS or BBS were added to each well to achieve the final serum concentrations stated above based on a final 250 µl volume. Bacteria to be tested were harvested from solid GC agar after 20–22 hrs growth, suspended in MEM, and 50 µl containing 1.5–2.5×10^3^ CFU were added to each well. Plates were incubated at 37°C in 7% CO_2_ for 1 hr, after which 50 µl of GCB were added and mixed. Two 50 µl aliquots were cultured on GC agar and the average number of CFU recovered was determined. The bactericidal_50_ titer was that concentration of antibody that resulted in a 50% reduction in the number of CFU recovered from wells that contained serum but no added test antibodies. Polyclonal rabbit antiserum against whole gonococci, which showed high level bactericidal activity against all Opa variants tested, was used as a positive control and antibodies that do not bind the target strain were used as negative controls. Heat-inactivated (HI) NHS and BBS were prepared by incubation at 56°C for 30 min, and were tested in parallel for each assay; none of the test or control antibodies had activity when HI serum was used. At least two independent experiments were performed for each test antibody, and the results were similar.

### Agglutination Assay

Agglutination titers were determined by the method of Pal et al [37]. Bacteria from primary subcultures of frozen stocks of Opa variants were suspended in PBS and passed through a 1.2 µm pore to remove aggregates. Filter suspensions were adjusted to an A_600_ of 0.4. Test antibodies or antisera were serially diluted 2-fold and 10 µL of each dilution were incubated with 10 µL of bacteria (∼5×10^6^ CFU) at 37°C in a microtiter plate for 45 min. Bacteria were incubated with the same dilutions of normal mouse sera (NMS) or affinity-purified polyclonal rabbit antibodies against heterologous HV_2_ loops in parallel. After incubation, 5 µL were spotted on a glass slide and stained with HEMA3 (Exaxol Corp). The average number of aggregates per at least three 40X fields was determined under light microscopy. Agglutination titers were defined as the highest dilution of test antibodies that caused greater than three times the number of aggregates seen in the same dilution of NMS or control antibodies. Comparisons between piliated and non-piliated variants of the same Opa type were also performed and identical results were obtained (data not shown).

### Passive Protection Experiments

Female BALB/c mice 6–8 weeks of age (National Cancer Institute, Frederick, MD) were treated with 1.5 mg water-soluble 17β-estradiol (Sigma) and antibiotics to promote susceptibility to *N. gonorrhoeae* as described [38]. In pilot experiments, 5–8 mice per group were inoculated vaginally with 10^3^, 10^4^, or 10^5^ CFU of predominantly OpaB-variants that were pre-incubated in PBS with 250 or 500 µg/ml of Ab_HV2BD linear_ or in PBS alone for 20 min at 37°C. In subsequent experiments with loop-specific affinity purified rabbit antibodies, bacteria (∼5×10^4^ CFU/ml) were preincubated in PBS with 250 µg/ml of Ab_HV2A_ _linear,_ Ab_HV2BD linear,_ or Ab_SV linear_ and 20 µl of the suspension (∼10^3^ CFU and 5 µg of antibodies) were inoculated vaginally into mice (n = 7–15 mice per group). In studies with mouse antisera against cyclic peptides, mice were inoculated with a 20 µl suspension containing 6×10^3^ CFU that were preincubated with Ab_HV2BD_ _cyclic_, Ab_SV_ _cyclic_, or NMS (final dilution of antiserum or NMS, 1∶30) (n = 10–11 mice per group). For all experiments, vaginal mucus was quantitatively cultured for *N. gonorrhoeae* daily for 3 days as described [32].

### Measurement of Antibody Duration

In separate experiments, the amount of topically applied antibody recovered from the vagina was measured over time by inoculating 23 untreated, 6 week-old female BALB/c mice vaginally with 20 µL of PBS containing 10 µg of affinity-purified rabbit polyclonal Opa-specific antibodies. The vaginas of 2–3 mice per time point were washed 3 times with 40 µL PBS and the three samples from each mouse were pooled (∼120 µL) and centrifuged at 13,000 rpm for 3 min. Supernatants were frozen at −20°C. Control samples were collected from 3 untreated mice. The concentration of rabbit IgG in murine vaginal washes was measured with the Rabbit IgG Quantitative Kit ELISA (Bethyl Laboratories). Animal experiments were conducted in the laboratory animal facility at USUHS, which is fully accredited by the Association for the Assessment and Accreditation of Laboratory Animal Care, under a protocol approved by the USUHS Institutional Animal Care and Use Committee.

### Adherence Assay

ME180 cervical epithelial cells (ATCC, Manassas, VA) were grown to near confluency in 24-well tissue culture plates in McCoy's 5A medium supplemented with 10% heat-inactivated fetal bovine serum (FBS) (Quality Biological Inc., Gaithersburg, MD) and 2.2 g/L sodium bicarbonate. Non-piliated OpaB-expressing bacteria were subcultured from the freezer and passed once to GC agar before being suspended in McCoy's 5A medium supplemented with 2.2 g/L sodium bicarbonate and 5 mg/L Fe(NO_3_)_3_ to an A_600_ of 0.07. Bacterial suspensions were diluted 1∶10 and pre-incubated for 5 min with mouse antisera against HV_2_ or SV cyclic peptides (test) or NMS (negative control) (final dilutions 1∶30 and 1∶100), or with 0.25 µg/mL or 2.5 µg/mL of Ab_HV2BD_ _linear_ (test) or Ab_HV2C_ _linear_ (negative control). Bacterial suspensions (500 µl) were applied to cells (multiplicity of infection, 10∶1) in triplicate wells. After 2 hrs at 37°C in 7% CO_2,_ monolayers were washed four times with PBS to remove nonadherent bacteria. Cells were lysed with 0.5% saponin (Sigma) and the number of cell-associated bacteria was determined by serial dilution and culture of the saponin-treated supsensions. Results are expressed as the number of cell-associated bacteria divided by the number of bacteria in the inoculum (% cell-associated). The average percent of cell-associated bacteria recovered from in test and control wells was calculated from three independent experiments that were each performed in triplicate. Standard error bars are shown.

### Statistical Analysis

A Fishers Exact test was used to compare the number of mice colonized in each experimental group in passive protection experiments. Differences in the number of gonococci recovered from mice and the recovery of cell-associated gonococci in tissue culture experiments were analyzed by the Student's t-test.
